# Prevalence of Clonal Complexes and Virulence Genes among Commensal and Invasive *Staphylococcus aureus* Isolates in Sweden

**DOI:** 10.1371/journal.pone.0077477

**Published:** 2013-10-09

**Authors:** Gunlög Rasmussen, Stefan Monecke, Ralf Ehricht, Bo Söderquist

**Affiliations:** 1 Department of Infectious Diseases, Örebro University Hospital, Örebro, Sweden; 2 Alere Technologies GmbH, Jena, Germany; 3 Institute for Medical Microbiology and Hygiene, TU Dresden, Dresden, Germany; 4 Laboratory Medicine, Clinical Microbiology, Örebro University Hospital Sweden, Örebro, Sweden; 5 Faculty of Medicine and Health, Örebro University, Örebro, Sweden; University Hospital Münster, Germany

## Abstract

*Staphylococcus aureus* encodes a remarkable number of virulence factors which may contribute to its pathogenicity and ability to cause invasive disease. The main objective of this study was to evaluate the association between *S. aureus* invasiveness and bacterial genotype, in terms of the presence of virulence genes and affiliation to clonal complexes. Also, the significance of different virulence genes, mainly adhesins, for the development of infective endocarditis was investigated.

DNA microarray technology was used to analyze 134 *S. aureus* isolates, all methicillin-susceptible, derived from three groups of clinically well-characterized patients: nasal carriers (n=46), bacteremia (n=55), and bacteremia with infective endocarditis (n=33).

Invasive isolates were dominant in four of the major clonal complexes: 5, 8, 15, and 25. Of the 170 virulence genes examined, those encoding accessory gene regulator group II (*agr* II), capsule polysaccharide serotype 5 (*cap*5), and adhesins such as *S. aureus* surface protein G (*sasG*) and fibronectin-binding protein B (*fnbB*) were found to be associated with invasive disease. The same was shown for the leukocidin genes *lukD/lukE*, as well as the genes encoding serine protease A and B (*splA/splB*), staphylococcal complement inhibitor (*scn*) and the staphylococcal exotoxin-like protein (*setC or selX*). In addition, there was a trend of higher prevalence of certain genes or gene clusters (*sasG, agr* II, *cap*5) among isolates causing infective endocarditis compared to other invasive isolates. In most cases, the presence of virulence genes was linked to clonal complex affiliation.

In conclusion, certain *S. aureus* clonal lineages harboring specific sets of virulence genes seem to be more successful in causing invasive disease.

## Introduction

*S. aureus* commonly colonizes the human skin and mucosal membranes without causing disease. However, it is also a well-known pathogen causing a broad spectrum of infections ranging from superficial skin infections to invasive disease such as life-threatening bacteremia and infective endocarditis (IE). An increased incidence of *S. aureus* bacteremia has been reported [1], and it constitutes the most common etiology of IE in Sweden with increased incidence in recent years (unpublished data from the Swedish national infective endocarditis quality register). The bacterium produces several virulence factors which may contribute to its invasive potential [2], including surface-associated adhesins such as MSCRAMMs (Microbial Surface Components Recognizing Adhesive Matrix Molecules) as well as secreted virulence factors like exotoxins and enzymes. Capsular polysaccharides and regulators may also contribute to pathogenicity [2,3]. Since *S. aureus* is a commensal and acts as an opportunistic pathogen, the predisposing factors of the host, together with virulence factors of the microorganism might play a significant role for invasiveness.

Previous studies have investigated the importance of *S. aureus* clonality for invasive disease [4,5], as well as associations between different virulence genes and *S. aureus* disease [6–12], though the results have shown some contradictions. MSCRAMMs are among the factors of interest, as they are known to have the capacity to bind extra-cellular matrix proteins such as fibrinogen, fibronectin, and elastin, all of which are potentially important for the invasive capacity of *S. aureus*. More specifically, the association of adhesins such as clumping factors (Clf) and fibronectin-binding proteins (FnbP) and development of IE has previously been examined, although mainly in experimental studies [13–15]. Still, additional clinical research is needed to investigate the significance of bacterial genotype for *S. aureus* disease.

The present study aimed to evaluate the potential association between *S. aureus* invasive disease and bacterial genotype in terms of the presence of genes encoding adhesins and other virulence factors as well as affiliation to clonal complexes (CCs). Furthermore, it sought to investigate whether the prevalence of certain MSCRAMM genes is associated with *S. aureus* IE. To achieve these aims, we used DNA microarray technology to analyze *S. aureus* isolates derived from three groups of clinically well-characterized patients: nasal carriers, bacteremia, and bacteremia with IE.

## Material and Methods

### Ethics statement

The study was conducted in accordance with the ethical guidelines of Declaration of Helsinki and was approved by the regional ethical committee in Örebro (Dnr 543/88) and Uppsala (Dnr 2011/3349). Written informed consent to participate was provided from all patients comprising the bacteremia group without IE (Dnr 543/88). Regarding the IE patients, the regional ethical committee in Uppsala approved the use of these clinical data entered in the Swedish quality register of infective endocarditis (Dnr 2011/3349). These IE patients were written informed that clinical data were recorded in a national quality register and could be used for research purposes.

Nasal isolates were obtained from swabs of anonymized patients intended for elective orthopedic surgery who were verbally informed regarding the purpose according to a Chairman decision of the regional ethical committee in Örebro 1992. For the IE patients and the anonymized patients whose nasal swabs were used, the regional ethical committee waived the need for written consent.

### Bacterial isolates

A total of 134 methicillin*-*susceptible *S. aureus* (MSSA) were included in the study: 46 nasal carriage and 88 bacteremic isolates. Within the bacteremia group, 33 isolates originated from patients with IE. The isolates were evaluated according to origin (nasal carriage, bacteremia with and without IE).

All isolates were derived from patients treated at Örebro University Hospital, and included in the study after ethical approval. Most of the bacteremic isolates (61/88) and corresponding clinical data were prospectively collected from hospitalized patients with a diagnosis of *S. aureus* bacteremia during 1988-1992 [16]. This group included 6 patients with concomitant IE. An additional 27 *S. aureus* blood isolates from patients with IE were also included. These isolates originated from patients who had been treated for *S. aureus* IE during 2008-2011 and registered in the Swedish national IE quality register, a national database recording relevant clinical data, diagnostic procedures, treatment, and outcome. Nasal carriage isolates were obtained from nasal swabs of patients intended for elective orthopedic surgery during 1992. For each patient, only one episode of *S. aureus* bacteremia was included, with the exception of one patient who had two separate episodes of bacteremia with genotypically different isolates.

The *S. aureus* isolates were identified by routine microbiological methods such as coagulase and DNase tests. Isolates were stored at -70°C in preservation medium (yeast extract; Difco Laboratories, Sparks, MD, USA; and horse serum added to trypticase soy broth; BBL, Sparks, MD, USA).

### Definitions

The diagnosis of *S. aureus* bacteremia was verified by at least one positive blood culture performed with the Bactec system (Becton Dickinson, USA)In order to evaluate the importance of different patient characteristics, the patients’ medical records were carefully surveyed for underlying conditions such as diabetes mellitusrenal insufficiency requiring hemodialysis or peritoneal dialysis, present malignant diseaseand immunosuppression due to treatment with corticosteroids, chemotherapyIE: infective endocarditisFnbP: fibronectin-binding proteinsMLST: multilocus sequence typingCCs: clonal complexesCP: capsular polysaccharidePIA: polysaccharide intercellular adhesionSTs: sequence types or immunosuppressive disease such as leukemia. Presence of indwelling catheters (e.g. central venous catheter), pacemakers, cardiac valves, prosthetic joint devices, and osteosynthesis material were recorded. In addition we registered hematogenous complications such as IE, acute osteomyelitis, septic arthritis, deep seated abscesses, and meningitis. IE was defined according to the modified Duke criteria [17]. 

### DNA microarray based genotyping

Genotyping was performed with the Alere StaphyType DNA microarray test (Alere Technologies GmbH, Jena, Germany), according to protocols and procedures described in detail elsewhere [18,19]. This DNA microarray includes 333 target sequences corresponding to approximately 170 distinct genes and their allelic variants, covering species markers, markers for the recognition of SCC*mec*, and capsule types as well as *agr* groups, resistance genes, exotoxins, MSCRAMM genes, and others. Primer and probe sequences have been published previously [19,20].

In short, monoclonal *S. aureus* cultures were grown on Columbia blood agar overnight at 37°C. Harvested cultures were enzymatically lysed using lysozyme, lysostaphin, and RNAse. DNA was prepared using commercially available spin columns (Qiagen, Hilden, Germany). The resulting RNA-free unfragmented DNA preparations were used as templates in a multiplexed primer elongation with only a single reverse primer per target. During that step, biotin-16-dUTP was incorporated into the amplicons. Labeled amplicons were hybridized to the microarray. After washing and blocking steps, horseradish-peroxidase-streptavidin conjugate was added. Following further incubation and washing, hybridizations were visualized using a precipitating dye. An image of the microarray was recorded using a designated reader (Alere Technologies GmbH, Jena, Germany). Normalized intensities of the spots were calculated based on their average intensities and further analyzed as described previously [20].

The assignment of isolates to CCs or sequence types (STs), as defined by multilocus sequence typing (MLST) [21], was determined by an automated comparison of hybridization profiles to a collection of reference strains previously subjected to MLST [19].

### Statistics

Proportions were compared with Fisher’s exact test, using version 17 of the SPSS software package. *P*-values <0.05 were considered statistically significant.

## Results

### Patient characteristics

Clinical data for patients with bacteremia, with and without IE, are shown in Table 1. No clinical data were available for the nasal carriage population. All 33 patients with IE fulfilled the criteria of definite IE according to the modified Duke criteria [17]. Of patients with IE, 19 of 33 (58%) had one or more factors predisposing for IE (underlying heart disease n=5; prosthetic heart valves n=3; previous history of IE n=4; injection drug use n=12). All patients except one were examined by echocardiography (28 transesophageal and 4 transthoracic). The diagnosis was confirmed by autopsy in two patients, one of whom did not undergo echocardiographic investigation. Twenty-three patients showed vascular embolization (osteomyelitis n=1; septic arthritis n=6; embolization to central nervous system/meningitis n=6; septic pulmonary infarcts n=11; skin and soft tissue n= 4). Eight patients underwent surgical intervention due to IE, and four patients relapsed shortly after the end of treatment or operation.

**Table 1:** Characteristics of patients with *S. aureus* bacteremia with or without IE.

**Clinical characteristics**	**Bacteremia IE n=33 (%)**	**Bacteremia non IE n=55 (%)**	**Bacteremia all n=88 (%)**
Median age	56 (range 24-91)	66 (range 10-91)	63 (range 10-91)
Sex (male)	18 (55)	34 (62)	52 (59)
Patients with ≥2 blood cultures positive for *S. aureus*	31 (94)	47 (85)	78 (89)
Immunosuppression^1^	3 (9)	5 (9)	8 (9)
Diabetes mellitus^2^	2 (6)	9 (16)	11 (13)
Malignant disease	2 (6)	7 (13)	9 (10)
IV drug abuse	12 (36)	1 (2)	13 (15)
Indwelling devices^3^	8 (24)	12 (22)	20 (23)
Hematogenous complications^4^	33 (100)	25 (45)	58 (66)
Acute osteomyelitis	1 (3)	14 (25)	15 (17)
Acute septic arthritis	6 (18)	9 (16)	15 (17)
Meningitis	1 (3)	2 (4)	3 (3)
Other CNS embolization	5 (15)	0 (0)	5 (6)
Mortality^5^	3 (9)	3 (5)	6 (7)

1 Treatment with corticosteroids, chemotherapy or immunosuppression due to leukemia

2 Treated with insulin or oral antidiabetics

3 Prescence of central venous catheter (n=2), pacemaker, cardiac valves, prosthetic joint devices or osteosynthesis material

4 IE, acute osteomyelitis, septic arthritis, deep seated abscesses or meningitis

5 Within 12 weeks after the initial positive blood culture

### Assignment to CCs and STs according to DNA microarray analysis

The assignment of 134 *S. aureus* isolates to CCs and STs is shown in Table 2. The isolates displayed 18 CCs/STs. Six CCs dominated and accounted for 113 of the 134 isolates (84%), each comprising 9 to 36 isolates. Four of the major CCs (5,8,15,25) included 46 isolates, 37 invasive and 9 carriage isolates, and this difference was statistically significant (p=0.012). Moreover, within CCS 5, 7 of 11 isolates (64%) originated from patients with IE. Counting on significance level, isolates within the four major CCs were combined, considering the relatively limited number in each CC. In contrast, *S. aureus* carriage within CC30 accounted for 18 of the 31 isolates (p=0.002). The 36 isolates in CC45 showed no difference between the isolates obtained from the carriage and invasive groups. The remaining 12 CCs/STs comprised 21 of 134 isolates (16%), each CC/ST represented by 1–5 isolates each. ST34 was analyzed separately from CC30 to which it belongs according to MLST based clustering because of distinct characteristics (absence of *cna*, presence of *seh*). Of the 2 isolates within ST34, both originated from IE patients.

**Table 2:** Distribution of CCs and ST.

**Clonal complex**	**Isolates No within CC**	**Nasal carriage (%)**	**Invasive (%)**	**Odds ratio (95% CI)**	**p-value^1^**
5	11^2^	1 (9)	10 (91)	3.0 (1.3-6.9)^3^	0.012^3^
8	9	2 (22)	7 (78)		
15	15	3 (20)	12 (80)		
25	11	3 (27)	8 (73)		
30	31	18 (58)	13 (42)	0.25 (0.11-0.57)	**0.002**
45	36	14 (39)	22 (61)	0.72 (0.32-1.6)	0.42
other^4^	21	5 (24)	16 (76)	na	na
Total	134	46 (34)	88 (66)		

1 Fisher’s exact test

2 7/11 isolates originated from patients with IE

3 Counted on CCS 5, 8, 15 and 25 together

4 ST34, CC1, CC9, CC12, CC20, CC22, ST49, CC50, CC59, CC97, CC182, CC395 na; not applicable

Bold figures; statistically significant

### *Agr* groups

The distribution of isolates among *agr* groups I-IV according to carrier status and invasive disease is shown in Table 3, along with affiliation to CCs. Affiliation to *agr* groups was strongly linked to bacterial clonality in that all isolates assigned to a specific CC clustered to the same *agr* group (Table 3). A total of 46% (62/134) of the MSSA isolates were assigned to *agr* group I and only 6 isolates were assigned to *agr* group IV. Neither group showed differences regarding the clinical origin of bacterial isolates. However, within *agr* group II, 26 of 31 isolates were of invasive origin and 13 of these were from patients with IE, but in *agr* group III, 18 of 35 isolates were carriage isolates. Thus, *agr* group II was associated with invasive disease (p=0.017), while *agr* group III was linked with carriage status (p=0.022).

**Table 3:** Distribution of *agr* groups.

***agr***	**Isolates n=134 (%)**	**Nasal carriage n=46 (%)**	**Invasive n=88 (%)**	**Odds ratio (95% CI)**	**p-value^1^**
I^2^	62 (46)	21 (46)	41 (47)	1.0 (0.51-2.1)	1
II^3^	31 (23)	5 (11)	26 (30)	3.4 (1.2-9.7)	**0.017**
III^4^	35 (26)	18 (39)	17 (19)	0.37 (0.17-0.82)	**0.022**
IV^5^	6 (4)	2 (4)	4 (5)	1.0 (0.19-5.9)	1

1 Fisher’s exact test

2 CC8, CC20, CC22, CC25, CC45 (except CC45 agr IV), CC59, CC97, CC182, ST 426

3 CC5, CC9, CC12, CC15, ST49

4 CC1, CC30, ST34

5 CC45 agr IV, CC50

Bold figures; statistically significant

### Surface-associated polysaccharides

**Table 4:** Presence of virulence genes.

**Virulence gene**	**Product**	**Nasal carriage n=46 (%)**	**Invasive n=88 (%)**	**Odds ratio (95%CI)**	**p-value^1^**
**Exopolysaccharides**					
*cap*5	capsular polysaccharide 5	8 (17)	31 (35)	2.6 (1.1-6.2)	**0.044**
*cap*8	capsular polysaccharide 8	38 (83)	57 (66)	0.39 (0.16-0.93)	**0.044**
*icaA,D,C*	polysaccharide intercellular adhesin	46 (100)	88 (100)		1
**MSCRAMM genes**					
*bbp*	Bone sialoprotein-binding protein	39 (85)	79 (90)	1.6 (0.55-4.55)	0.41
*clfA, clfB*	Clumping factors A and B	46 (100)	88 (100)		
*cna*	Collagen binding adhesin	37 (80)	43 (49)	0.23 (0.10-0.54)	**<0.0001**
*ebh*	Cell wall associated fibronectin-binding protein	44 (96)	86 (98)	2.0 (0.27-14)	0.61
*ebps*	Cell surface elastin-binding protein	46 (100)	88 (100)		1
*eno*	Enolase	46 (100)	88 (100)		1
*fib*	Fibrinogen binding protein	46 (100)	88 (100)		1
*fnbA*	Fibronectin-binding protein A	46 (100)	88 (100)		1
*fnbB*	Fibronectin-binding protein B	27 (59)	71 (81)	2.9 (1.3-6.5)	**0.008**
*map*	Major hisocompatibility complex class II analog protein	46 (100)	87 (99)	1.5 (1.4-1.7)	1
*sasG*	*S. aureus* surface protein G	8 (17)	38 (43)	3.6 (1.5-8.6)	**0.004**
*sdrC*	Serine-aspartate repeat protein C	46 (100)	88 (100)		1
*sdrD*	Serine-aspartate repeat protein D	40 (87)	81 (92)	1.7 (0.55-5.5)	0.37
*vwb*	Van Willebrand factor binding protein	46 (100)	88 (100)		1
**Exotoxins**					
**Hemolysins**					
*hla*	α-toxin	46 (100)	86 (98)		0.55
*hlb*	β-toxin	39 (85)	71 (88)	0.80 (0.30-2.1)	0.81
*hld*	δ-toxin	46 (100)	88 (100)		1
hl^2^	Putative protein similar to hemolysin	46 (100)	87 (99)		1
*hlIII* ^3^	Channel protein, hemolysin III family protein	45 (98)	87 (99)		1
**Leukocidins**					
*lukF, lukS, hlgA*	γ-toxin	46 (100)	88 (100)		1
*lukF-PV, lukS-PV*	Panton-Valentine leukotoxin	0 (0)	1 (1)		1
*lukD, lukE*	Leukocidin D, E component	12 (26)	46 (52)	3.1 (1.4-6.8)	**0.006**
*leukocidin homologue gene* ^4^	Leukocidin homologue family protein	46 (100)	88 (100)		1
**Exfoliative toxins**					
*etA*	Exfoliative toxin A	3 (7)	4 (5)		0.69
*etB*	Exfoliative toxin B	0 (0)	0 (0)		1
*etD*	Exfoliative toxin D	3 (7)	8 (9)		0.75
**Enterotoxins**					
sea	Staphylococcal enterotoxin A	10 (22)	16 (18)	0.80 (0.33-1.9)	0.65
*sea* (*N315*)^5^	Staphylococcal enterotoxin A, allele from N315	2 (4)	12 (14)		0.14
*seb*	Staphylococcal enterotoxin B	2 (4)	7 (8)		0.72
*sec+sel*	Staphylococcal enterotoxin C+L	7 (15)	12 (14)	0.88 (0.32-2.4)	0.80
*sed+sej+ser*	Staphylococcal enterotoxin D+J+R	3 (7)	9 (10)		0.55
*see*	Staphylococcal enterotoxin E	0 (0)	0 (0)		1
*egc-cluster* ^6^	Staphylococcal enterotoxin G+I+M+N+O+U	40 (87)	61 (69)	0.34 (0.128-0.89)	**0.034**
*seh*	Staphylococcal enterotoxin H	1 (2)	4 (5)		0.66
*sek+seq*	Staphylococcal enterotoxin K+Q	0 (0)	4 (5)		0.30
*tst1*	Toxic shock syndrome toxin (TSST)-1	15 (33)	15 (17)	0.43 (0.19-0.97)	0.050
**Enzymes**					
*aur*	Aureolysin	46 (100)	88 (100)		1
*splA* ^7^, *splB* ^8^	Serine protease A, B	12 (26)	46 (55)	3.4 (1.6-7.4)	**0.002**
*splE*	Serine protease E	29 (63)	50 (57)	0.77 (0.37-1.6)	0.58
*sspA* ^9^	Glutamyl endopeptidase	46 (100)	88 (100)		1
*sspB* ^10^+*sspP* ^11^	Staphopain B, A	46 (100)	88 (100)		1
*sak*	Staphylokinase	38 (83)	73 (83)	1.0 (0.40-2.6)	1
*chp*	Chemotaxis inhibitory protein	38 (83)	69 (78)	0.77 (0.31-1.9)	0.65
*scn*	Staphylococcal complement inhibitor	41 (89)	86 (98)	5.2 (0.98-28)	**0.047**
**Miscellaneous genes**					
*edinA+C*	Epidermal cell differentiation inhibitor A+C	0 (0)	0 (0)		1
*edinB*	Epidermal cell differentiation inhibitor B	3 (7)	8 (9)		0.75
*setC* ^12^	Staphylococcal exotoxin-like protein	25 (56)	70 (81)	3.3 (1.5-7.3)	**0.004**
isdA	Transferrin binding protein	46 (100)	88 (100)		1

1 Fisher’s exact test

2 Locus tag SACOL0921, GenBank CP000046.1: Position 927776-928816

3 Locus tag SACOL2160, GenBank CP000046.1: Position 2239231-2239914

4 Locus tag SACOL2004, GenBank CP000046.1: Position 2064956-2065972, Locus tag SACOL2006, GenBank CP000046.1: Position 2065994-2067049

5 Also known as enterotoxin sep

6 5 isolates belonging to CC50 showed a partial deletion of the locus egc-cluster missing seg

7 Locus tag SACOL1869, GenBank CP000046.1: Position 1920785-1921501

8 Locus tag SACOL1868, GenBank CP000046.1: Position 1919938-1920660

9 Locus tag SACOL1057, GenBank CP000046.1: Position 1063016-1064026

10 Locus tag SACOL1056, GenBank CP000046.1: Position 1061753-1062934

11 Locus tag SACOL1970, GenBank CP000046.1: Position 2034319-2035485

12 Locus tag SACOL0442, GenBank CP000046.1: Position 445958-446569. Two isolates with ambiguous results not included.

Bold figures; statistically significant

The distribution of genes encoding exopolysacharides, MSCRAMMs, exotoxins, enzymes and other miscellaneous genes are shown in Table 4.

**Figure 1:**
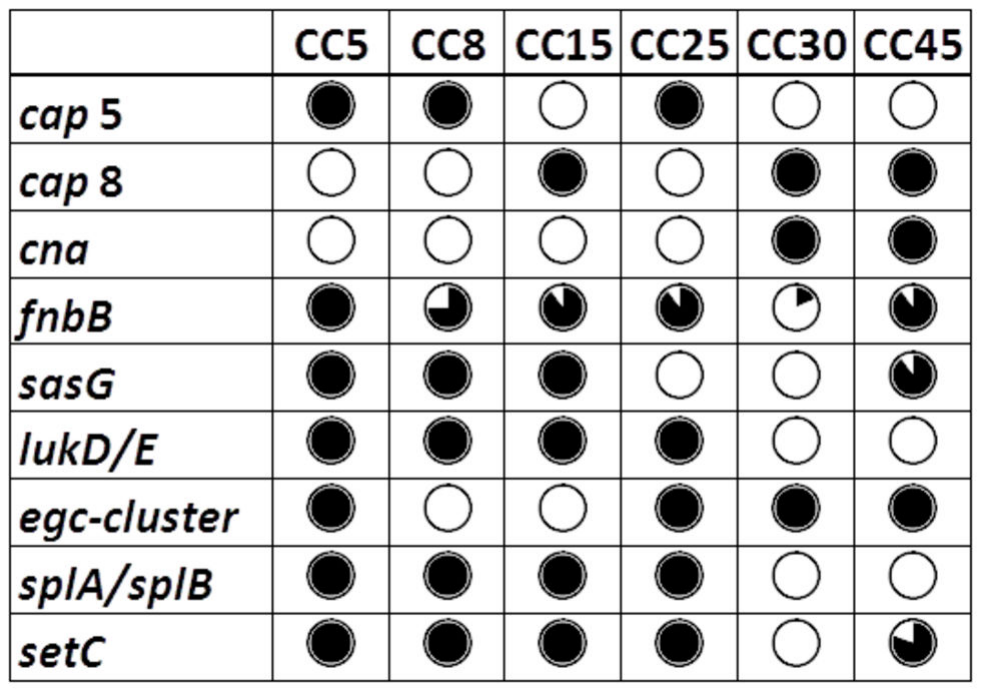
Distribution of genes associated with invasive disease or nasal carriage status in relation to CCs. Black circle indicates that all isolates within the CC harbor the gene and white circle that none of the isolates does. A divided circle indicates variable gene presence within the CC. In most cases the presence of virulence genes was linked to CC affiliation.

All isolates carried capsular polysaccharide (CP) serotype 5 (29%) or 8 (71%); no CP1 was found. There was an association between invasiveness and CP5 (p=0.044); 15 of the 39 CP5 isolates (38%) were from patients with IE. The presence of *cap* genes was in accordance with affiliations to CCs (Figure 1), but there were no correlation between *cap* genes and *agr* groups.

All isolates harbored the genes encoding polysaccharide intercellular adhesion (PIA); *ica*A, *ica*C, and *ica*D.

### MSCRAMMs

Genes encoding 15 different MSCRAMMs were analyzed (Table 4), and two of these (*fnbB* and *sasG*) were more prevalent among invasive isolates than carriage isolates (p=0.008 and p=0.004, respectively), while the converse was observed for *cna* (p=<0.0001). Carriage of *sasG* and *cna* was in concordance with CC affiliation (Figure 1). The genes *bbp*, *ebh*, *map*, and *sdrD* were found in most isolates (88-99%), regardless of clinical origin, while the remaining genes (*clfA*, *clfB*, *ebpS*, *eno*, *fib*, *fnbA*, *sdrC*, and *vwb*) were present in all isolates. In addition, *bap*, encoding a biofilm-associated protein was abscent in all isolates.

### Genes encoding toxins

The hemolysin genes *hla, hld, hl*, and *hlIII* (loci and gene positions in Table 4) were present in all isolates except one *hla*-negative ST49 isolate and two *hlIII*-negative isolates within CC22. The *hlb* gene was found in 110/134 isolates (82%), with no difference between the invasive and the nasal carriage group. Of leukocidins, only one isolate carried the PVL-encoding genes (*lukF-PV*, *lukS-PV*). However, *lukD*/*lukE* were more prevalent among invasive isolates (46/88) compared to nasal carriage isolates (12/46), and were thus significantly associated with invasive disease (p=0.006). Genes encoding gamma-toxin (*lukF*/*lukS*/*hlgA*) as well as leukocidin homologue family protein (loci and gene position in Table 4) were ubiquitous.

Genes encoding exfoliative toxins were absent (*etB*) or rare (*etA* 5%; *etD* 8%), and all *etD*-positive isolates were assigned to CC25. The *tst*1-gene was found in 22% of isolates, and was more prevalent among nasal carriage isolates compared to invasive isolates (p=0.005).

Of enterotoxin genes, those within the *egc* cluster (*seg*+sei+*sem*+*sen*+*seo*+*seu*) were the most frequent (64%) ones, being linked to bacterial clonality (Figure 1) and significantly associated with carriage isolates (p=0.034). The remaining enterotoxin genes were found in 0–19% of the isolates with no difference in prevalence with regard to clinical origin.

The presence of genes encoding proteases, *splA* and *splB* (loci and gene position in Table 4) was associated with invasive isolates (p=0.002), but there was no difference concerning *splE*. *SspA*, *sspB*, and *sspP* (loci and gene position in Table 4) were ubiquitous among isolates. *SetC* (also known as *selx*, locus and gene position in Table 4) was also found to be associated with invasive disease (p=0.004). Genes encoding HLB-converting phages (*sak*, *chp*, *scn*) were present in 80–95% of isolates but genes encoding epidermal cell differentiation inhibitors were absent (*edinA*, *edinC*) or rare (*edinB*; 8%). All isolates harbored the gene *isdA*, encoding transferrin-binding protein.

### Genes associated with invasive disease

A further comparison between invasive non-IE isolates and IE isolates was performed in virulence genes which demonstrated a significant association with invasive disease (Table 5).

**Table 5:** Comparison between invasive non IE isolates and IE isolates in virulence genes associated with invasive disease.

**Virulence gene**	**Nasal carriage n=46 (%)**	**Invasive non IE n=55 (%)**	**Invasive IE n=33 (%)**
*agr* II	5 (11)	13 (24)	13 (39)
*cap*5	8 (17)	16 (29)	15 (45)
*fnbB*	27 (59)	47 (85)	24 (73)
*sasG*	8 (17)	18 (33)	20 (61)
*lukD/lukE*	12 (26)	28 (51)	20 (61)
*splA, splB*	12 (26)	28 (51)	20 (61)
*setC*	25 (56)	44 (80)	26 (79)

## Discussion

*S. aureus* encodes a remarkable number of surface-associated as well as extracellular virulence factors. However, previous studies have shown contradictory results regarding the importance of these different virulence genes for invasiveness. Based on 134 MSSA isolates from three well-characterized patient populations, the aim of the present study was to evaluate the potential association between *S. aureus* disease and bacterial genotype with a focus on clonality and genes encoding MSCRAMMs. We used DNA microarray based analysis, which offers the great advantage of a simultaneous and fast analysis of a large number of virulence genes as well as assignment to clonal lineages at a very reasonable cost compared to multiple PCRs or whole genome sequencing.

The *S. aureus* isolates were distributed between six major CCs, mainly in concordance with previous studies from Europe [5,10,22] and the USA [4]. CC30 and CC45 comprised the largest number of isolates. Within the other four major CCs (5, 8, 15, and 25), invasive isolates dominated. In contrast, some previous studies have failed to show an association between invasive disease and clonality [5,9]. By studying less heterogeneous *S. aureus* infections, Fowler et al [4] could show an association between invasive disease with hematogenous complications and isolates within CC5 and CC30. The results of Fowler et al are in accordance with our results for CC5 but not for CC30. However, in their study it was the MRSA isolates within CC30 that were significantly associated with invasiveness. Our study only involved MSSA isolates, and CC30-MRSA are rare to virtually non-existent in Sweden. Thus epidemiological differences between the places where the studies were performed may explain this apparent discrepancy. Of isolates assigned to CC5, the majority originated from patients with IE. Although the total number of isolates within CC5 was limited, this result may suggest an association between CC5 and serious invasive *S. aureus* disease.

Due to polymorphisms in the gene encoding an autoinducing peptide in the *agr* locus, the *S. aureus* isolates are assigned to *agr* groups I to IV. As described in previous studies, the *agr* groups are strongly linked to bacterial clonality [8,19], although both the present study and previous work [19] showed that isolates within the same *agr* group may belong to genetically diverse CCs. In our study, isolates within *agr* II were significantly associated with invasive disease, and 50% of invasive isolates within *agr* II originated from patients with IE. Since *agr* II included isolates assigned to CC5 and CC15, and invasive isolates dominated within these clones, it is not possible to conclude whether this association is a consequence of clonality or assignment to *agr* group. A previous study by Jarraud et al also showed a positive relationship between *agr* II and IE [8].

As expected, all isolates carried CP serotype 5 or 8 [23]. Among the isolates that displayed CP5, invasive isolates dominated which is consistent with a previous study in which a CP5-positive strain in a mouse model showed a higher bacteremia level as well as enhanced capacity to persist in the bloodstream, compared to a CP8-positive strain [24]. It is also worth noting the even stronger correlation between CP5 and IE. However, as for *agr* group, CP type is linked to the affiliation to CCs, and so the association between CP5 and invasive disease as well as IE could be a consequence of clonality. The absence of correlation between capsule serotypes and *agr* groups is also in line with results in a previous study [19].

Due to the capacity of MSCRAMMs to bind extracellular matrix proteins, the significance of MSCRAMM genes for invasive disease and IE has been thoroughly examined previously [7,11–15,25]. The present study showed a significant association between the presence of *fnbB* as well as *sasG* and invasive disease, in accordance with previous studies [26,27]. There was also a trend towards a higher prevalence of *sasG* among isolates from IE patients compared to other invasive isolates. The presence of *sasG*, but not *fnbB*, was in accordance with affiliation to CCs.

The results are however difficult to interpret, due to the overlapping properties of many adhesins. Besides binding fibronectin, both FnbA and FnbB have the capacity to bind elastin; although unlike FnbA, FnbB has not been shown to promote platelet aggregation [28] or bind fibrinogen. The overall capacity to bind fibronectin is not crucial based on whether the isolate carries one or both *fnb* genes [27]. This may indicate that the additional role of FnbB, such as binding elastin, may be important for *S. aureus* invasive capacity. The lack of evidence for FnbB promoting platelet aggregation is consistent with our findings, which showed no difference in *fnbB* gene prevalence between the invasive non-IE isolate group and the IE isolate group.

The SasG protein shows opposing characteristics; it prevents adhesion to extracellular matrix components such as fibronectin, cytokeratin 10, and IgG, while at the same time it promotes adhesion to nasal epithelial cells as well as biofilm formation [29]. The gene *sasG* shares sequence similarity with a plasmin sensitive protein encoded by the gene *pls*, in which mutation has been correlated with reduced invasion of host cells [30]. It is thus plausible that *sasG* also plays an important role for *S. aureus* invasiveness. Unexpectedly, and contrary to previous studies [7,31,32], *cna* was more common in nasal carriage isolates than in invasive isolates. An explanation for this finding could be that *cna* is also a CC-associated marker. Since CC30 is overrepresented among carrier isolates and since all CC30 isolates also carry *cna*, the apparent association of this gene with carriage might be a function of the clonality of the strains in question. Furthermore, *cna* is the most important collagen-binding adhesin protein of *S. aureus* [33], and this feature may also be important for adherence to the nasal epithelial cells.

Several other MSCRAMM genes shown to be important for invasiveness and development of IE are highly conserved in the *S. aureus* genome [34]. This is in accordance with our results, where all isolates harbored the genes *clfA*, *clfB*, *fnbA*, and *sdrC*. On the other hand, *bap*, which encodes a biofilm-associated protein, was absent in all isolates, which is consistent with the fact that this gene has so far only been reported in animal strains [35].

As expected, *hla* was present in almost all isolates, with some occasional exceptions probably due to single mutations. Similarly, *hlb* was also present at high frequency, though its function depends on whether the *hlb*-converting prophage is integrated or not. In contrast to hemolysin genes, those encoding exfoliative toxins were rare, which was expected since patients with exfoliative staphylococcal disease were not specifically included in the present study. *Egc* was the most prevalent among enterotoxin genes, and as shown before linked to certain CCs and negatively associated with invasive disease [10,36]. Among leukocidins, the prevalence of *lukD*+*lukE* was high, linked to CCs and significantly associated with invasive disease, also shown by Eiff et al [37]. The *splA*/*splB* genes were significantly associated with invasive disease, as well as *setC*.

A limitation of our study is that we have merely investigated the presence of genes harbored by *S. aureus*, rather than their expression, and that analysis was restricted to those alleles of genes covered by the DNA microarray. Moreover, specific patient-related risk factors such as immunosuppression, intravenous drug use, and indwelling medical devices may predispose for invasive *S. aureus* disease. However, we included isolates from patients with these risk factors, as it might be a reason why not all patients with predisposing medical conditions get invasive disease. Conversely, a major advantage of the study is the well-characterized patient population.

In conclusion our study indicates that invasive *S. aureus* isolates are related to certain CCs. We also found a significant association between invasiveness and genes encoding CP type as well as specific MSCRAMMs such as *fnbB* and *sasG*. Moreover, our results suggest a trend toward even higher prevalence of certain virulence genes among isolates causing IE compared to other invasive isolates. However, in most cases the presence of virulence genes was linked to CC affiliation. It is also known that certain clonal lineages carry specific sets of virulence genes [20]. Even though *S. aureus* isolates from all CCs can cause invasive disease, it is reasonable to assume that certain *S. aureus* clonal lineages harboring specific sets of virulence genes are more successful at causing an invasive disease.

Against the background of increased life expectancy, implementation of more advanced medical interventions, and the emergence of antibiotic resistance, continuous surveillance of the incidence of *S. aureus* bacteremia is important. There may be continuous increase in the incidence of *S. aureus* bacteremia as well as changes in the structure and composition of the bacterial population. A study to further investigate any changes in the molecular epidemiology of invasive *S. aureus* isolates over the last 3 decades is currently ongoing.
