# Correction: Prevalence of Clonal Complexes and Virulence Genes among Commensal and Invasive *Staphylococcus aureus* Isolates in Sweden

**DOI:** 10.1371/journal.pone.0099097

**Published:** 2014-05-27

**Authors:** The PLOS ONE Staff

Several errors were introduced during the typesetting process. The publisher apologizes for these errors.

Text was mistakenly added to the Definitions section of the Materials and Methods. The correct paragraph is, “The diagnosis of *S. aureus* bacteremia was verified by at least one positive blood culture performed with the Bactec system (Becton Dickinson, USA). In order to evaluate the importance of different patient characteristics, the patients’ medical records were carefully surveyed for underlying conditions such as diabetes mellitus, renal insufficiency requiring hemodialysis or peritoneal dialysis, present malignant disease, and immunosuppression due to treatment with corticosteroids, chemotherapy, or immunosuppressive disease such as leukemia. Presence of indwelling catheters (e.g. central venous catheter), pacemakers, cardiac valves, prosthetic joint devices, and osteosynthesis material were recorded. In addition we registered hematogenous complications such as IE, acute osteomyelitis, septic arthritis, deep seated abscesses, and meningitis. IE was defined according to the modified Duke criteria [17].”

In the Surface-associated polysaccharides section of the Results, the first paragraph is mistakenly included in the footnote of Table 4. The first paragraph of the Surface-associated polysaccharides section is, “The distribution of genes encoding exopolysacharides, MSCRAMMs, exotoxins, enzymes and other miscellaneous genes are shown in Table 4.”
